# Patterns of Alcohol Use Among Italian Young Adults Before and During a COVID-19 Lockdown: A Latent Class Analysis Study

**DOI:** 10.1007/s10935-022-00675-2

**Published:** 2022-03-19

**Authors:** Giovanni Aresi, Angela Sorgente, Michael J. Cleveland, Elena Marta

**Affiliations:** 1 Psychology Department, Università Cattolica del Sacro Cuore, Largo Gemelli 1, 20123 Milan, Italy; 2 CERISVICO Research Centre on Community Development and Organisational Quality of Life, Via Trieste 17, 25121 Brescia, Italy; 3 Department of Human Development, Washington State University, 516 Johnson Tower, Pullman, WA 99164 USA

**Keywords:** Alcohol use, Drinking patterns, Young people, COVID-19, Lockdown, Person-centered approach, Italy

## Abstract

Two not mutually exclusive theories explain the effects of the COVID-19 pandemic on alcohol use. The *Availability hypothesis* contends that reduced opportunities to drink due to the closure of outlets and consumption sites should lead to decreases in alcohol use, whereas the *Stress and Coping hypothesis* argues that those exposed to stressful situations may increase drinking. The primary aim of this study was to examine changes—separately by gender—in the prevalence of drinking patterns among Italian young adults (18–34 years) before and during a COVID-19 lockdown. Study design was a repeated cross-sectional study, whereby data collected in 2015 and 2020 from nationally representative samples were analyzed. Latent class analysis identified five, fully invariant for women and partially invariant for men, drinking pattern classes among both cohorts: *current non-drinkers* (CND), *weekend risky* (WRD) and *weekend non-risky drinkers* (WnRD), *daily non-risky* (DnRD) and *daily risky drinkers* (DRD). In support of the *Availability hypothesis*, increases in abstaining and moderate drinking women and men were observed from 2015 to 2020. Concomitantly, among men only there were also increases in the prevalence of patterns characterized by risky drinking, coping drinking motives and related harm (*Stress and Coping hypothesis*). The pandemic and the three-tier lockdown imposed by the Italian government likely reduced overall alcohol use in the general population who drink moderately. However, there was a substantial increase in the prevalence of a small but significant group of men who drank daily and heavily to cope. Outreach and prevention efforts should target primarily this group, but also consider the opportunities that the exceptional circumstances of a quarantine offer to any individuals to reshape their lifestyle and health-related behaviors.

**Supplementary Information:**

The online version contains supplementary material available at 10.1007/s10935-022-00675-2.

## Introduction

The COVID-19 outbreak has dramatically affected the life of young people throughout the world, as nations implemented social distancing, isolation measures and restrictions to social life and to the operations of drinking venues in the attempt to reduce the spread of the virus. These measures are likely to have had an impact on several outcomes, including alcohol use. Alcohol use and misuse is one area of special concern for policymakers, practitioners, and academics, and it is therefore important to develop a nuanced understanding of the ways in which drinking patterns were affected by the health emergency. As the global pandemic continues, this knowledge is important as prevention scientists identify high-risk groups that may need targeted support and develop interventions accordingly.

There are two not mutually exclusive mechanisms and accompanying hypotheses to understand the effects of the pandemic on alcohol use (Rehm et al., 2020). The *Availability hypothesis* contends that reduced opportunities to drink due to social distance measures and the closure of outlets and consumption sites should lead to decreases in alcohol use in the general population and especially among lighter drinkers. The *Stress and Coping hypothesis* argues that those exposed to stressful situations, such as social isolation and socioeconomic insecurity, may experience mental health issues, which may contribute to increases in alcohol use as a dysfunctional coping strategy (i.e., self-medication). The tendency to self-medicate can be further exacerbated because traditional support mechanisms (i.e., social support and community services for treatment) around alcohol reduction may have become less accessible during the lockdown (Nicholls & Conroy, 2021).

Globally, findings of studies on adult populations in Australia, Canada, the U.S.A., and Europe offer support to each hypothesis. In Sallie et al. (2020) international study, drinking behaviors decreased overall during quarantine, though 36% of the sample reported an increase in alcohol use. Garnett et al. (2021) found about the same proportion of UK adults (25%) reporting drinking more and less alcohol than usual during the COVID-19 related lockdown with those younger, female, of high socioeconomic position, suffering from an anxiety disorder, and stressed about finances or COVID-19 to be at greater risk. Other studies in the UK, Norway and Canada indicate that increased alcohol use and misuse were associated with psychological, social and economic stress and vulnerability (Alpers et al., 2021; Naughton et al., 2021; Wardell et al., 2020).

Collectively, these studies provide evidence for non-univocal changes in drinking behavior during the pandemic. However, there is currently limited evidence on the effects on the young adult population. It is likely that COVID-19 restrictions may have had a greater impact on younger drinkers who have a richer social life outside their home, which often includes drinking at parties and public venues (White et al., 2020). This was confirmed by early evidence from two Australian studies that found young people reporting greater decreases in consumption during the pandemic (Callinan, Mojica-Perez, et al., 2021; Callinan, Mojica-Perez, et al., 2021; Callinan, Smit, et al., 2021; Callinan, Smit, et al., 2021).

For this reason, the *Availability hypothesis* may be particularly suitable for understanding the impact of the pandemic on this age cohort. For example, decreased alcohol use has been found among U.S. college student samples experiencing reduced opportunities to socialize and greater parental control as they moved back with parents (Graupensperger et al., 2021). On the other hand, young people may be stressed by the disruptions to their lives caused by the pandemic and the most vulnerable may acutely suffer as a result. In a U.S. study, symptoms of depression and anxiety due to university closings were linked to greater increases in alcohol consumption (Lechner et al., 2020). In Europe, a study of German young adults demonstrated that 24.4% reported a decrease in binge drinking while 5.4% reported an increase (Busse et al., 2021). These results suggest a rough estimate of the relative proportion of the young adult population who may respond to the pandemic consistent with either the *Availability* and *Stress and Coping hypotheses*.

Despite convergence in the amount of alcohol consumed by men and women, women generally continue to report lower levels of alcohol use than men (Kuntsche et al., 2011; Wilsnack et al., 2009). Theory and research suggest that moderate drinkers will consume less alcohol as an effect of the health emergency; thus, in general, one may expect that women responded to the pandemic by consuming even less alcohol than pre-pandemic. At the same time, pandemic-related stressors may represent unique challenges to the life of early adults, and affect alcohol use differently by gender as coping drinking motives are more closely connected to binge drinking among men than women (Peltier et al., 2019; Temmen & Crockett, 2020). To this date, however, the evidence on the gendered stress—alcohol misuse relation during the pandemic is mixed: a study reported that women highly stressed by the pandemic increased their alcohol use and reported similar levels compared to men (Rodriguez et al., 2020), though another study did not confirm such result (Schmits & Glowacz, 2021).

In November 2020, as the second COVID-19 wave was approaching, the Italian government established a three-tier scheme (yellow, orange, and red) in an attempt to avoid a second national lockdown (Governo Italiano, 2020). Italy’s 19 regions and two autonomous provinces were assigned to tiers on a weekly basis based on epidemiological data and the burden on the healthcare system (e.g., saturation of intensive care units). A 10 p.m. curfew was introduced and limitations to business operations including of alcohol outlets (e.g., opening hours) were enforced across all tiers. On-premise, but not off-premise and delivery, consumption was forbidden, except under the yellow tier between 5:00 am and 6:00 pm. Differently than in other countries, alcoholic beverages were included in the list of essential goods and services and were sold across a variety of sites including supermarkets as it is in usual times. In sum, Italy's response to the pandemic in November 2020 may have at least in part determined differences in drinking patterns across regions depending on the tier they were assigned to.

There are three key novel aspects to this study. First, the use of data collected prior and during the pandemic from nationally representative samples overcomes key methodological limitations of alcohol use measurement and to result generalizability. Besides a few exceptions (e.g., Garnett et al., 2021), most studies used convenience samples and measured alcohol use cross-sectionally asking respondents to assess their alcohol consumption during and prior to the pandemic (Busse et al., 2021), which put into question the generalizability and validity of findings (i.e., susceptibility to distortion and recall bias). Second, we used a different approach to data analysis, the person-centered approach (e.g., Latent Class Analysis—LCA), which consists of a classification system that groups individuals into distinct subgroups or typologies (i.e., drinker profiles) (see, among others, Collins & Lanza, 2010). Once developed, these typologies can be used to examine changes in class prevalence and transition probabilities across time and cohorts (Aresi et al., 2021; Cleveland et al., 2012). Analyzing groups of individuals also allows one to simultaneously test both the *Availability* and *Stress and Coping hypotheses* on different subgroups identified in the classification. Third, to our knowledge, this is the first published study that uses samples from a Southern European country. Characteristics of these “wet” cultures (Aresi et al., 2018, 2020; Beccaria, 2010; Room, 2007), such as more frequent but moderate alcohol use, and the mild weather and relaxed alcohol policies (which in turn, make drinking in the open air in public places very common among youth) (Calafat et al., 2011), may determine different responses to the health emergency as compared to other countries.

The primary aim of this study was to examine changes—separately by gender—in the prevalence of drinking patterns among Italian young adults (18–34 years) before and during a COVID-19 lockdown. We also examined the associations between membership to drinker profiles and lockdown tiers.

Based on findings of previous studies using LCA indicating that drinker class solutions tend to be relatively stable across gender and time (Aresi et al., 2021; Cleveland et al., 2012), we expected the number and characteristics of the classes will be broadly the same across the 2015 and 2020 cohorts. In addition, we tested the following hypotheses:

1. There has been an increase in the prevalence of profiles characterized by abstention along with a decrease in moderate drinking patterns (i.e., those that do not include risky drinking behaviors);
2. Among men but not women, there has been an increase in the prevalence of high-risk drinking patterns characterized by drinking to cope;
3. Living in stricter lockdown tiers would be associated with belonging to profiles that are characterized by abstention and moderate drinking.

## Methods

Study design was a repeated cross-sectional study. We analyzed data collected from nationally representative samples of Italian young adults in 2015 and during the November 2020 COVID-19 lockdown.

### Data

This study involves the secondary analysis of data collected by the ©Osservatorio Giovani of the Istituto Toniolo di Studi Superiori (Milan, Italy). Data were collected from nationally representative samples of Italian young adults (18 to 34 years old) in two cohorts: December 2015 and December 2020 during the second COVID-19 lockdown in Italy. Sampling and Computer Assisted Web Interview (CAWI) data collection was conducted by Ipsos s.r.l. Each cohort data is weighted to account for the non-random nature of the sample and guarantee sample representativeness in respect to several socio-demographic characteristics including gender, age-range, educational level, occupation status, and geographic area (Istituto Nazionale di Statistica, 2015, 2020). In both samples, lifetime abstainers (2015: N = 521; 2020: N = 264) were excluded from the analyses, resulting in analytical samples of 5950 and 1736 participants in 2015 and 2020, respectively. Table 1 displays weighted socio-demographic characteristics of participants by cohort.

**Table 1:** Participants' characteristics by cohort (weighted)

	Proportion^*^	
	2015	2020
Gender (female)	51.5	48.7
Mean age (*SD*)	25.7 (4.740)	26.9 (4.743)
Occupation		
Student	38.3	34.8
Worker	42	47.8
Unemployed/NEET**	19.2	17.4
Education		
University degree	18.5	22.2
High school diploma	50.5	50.6
Other	31	27.2
Residence		
Northwest	25	24.8
Northeast	17	17.8
Centre	19	19.2
South***	38.8	38.2

*Only proportions are displayed because data are weighted

**Not in Education, employment or training

***Includes Sicily and Sardinia

This study was reviewed and approved by the ©Osservatorio Giovani of the Istituto Toniolo di Studi Superiori. The study was conducted in accordance with the Declaration of Helsinki. All participants provided their written informed consent to participate in this study.

### Measures

In both surveys, participants were asked if they ever had any alcohol. Next, they indicated the number of drinks consumed each day of a typical week in the last month (three months in 2015), using the Daily Drinking Questionnaire (Collins et al., 1985), how many times during the past month they had gotten drunk, and the number of times they had consumed four (females) or five (males) or more drinks within two hours. Examples of drinks (e.g., a 250 ml beer) containing approximately 10 g pure ethanol were presented. These ten indicators of drinking were dichotomized (0 = no; 1 = yes): (1) past month alcohol use; (2) past month drunkenness; (3) past month Heavy Episodic Drinking (HED); and (4–10) DDQ indicators of alcohol use for each day of a typical week.

In 2015, participants also completed a three-item measure of coping drinking motives (Drinking Motive Questionnaire Revised Short Form, DMQ-R SF; α = 0.825) (e.g., *“How often did you drink because it helps you when you feel depressed or nervous?”*) (0 = never, 1 = sometimes; 2 = almost always) (Mazzardis et al., 2010), and seven adapted from the Brief Young Adult Alcohol Consequences Questionnaire (BYAACQ; Kahler et al., 2008). The summed score of the BYAACQ dichotomous items (0 = no, 1 = yes) represented the total number of consequences experienced in the previous 30 days (e.g., *I have had a hangover (headache, sick stomach) the morning after I had been drinking*) (BYAACQ; Kahler et al., 2008). For a description of the items, see Aresi et al. (2018).

Participants of the 2020 cohort were assigned to lockdown tiers (0 = yellow; 1 = orange; 2 = red) depending on their region of residence allocation during the four weeks prior to data collection. In those cases when regions changed tier during that period, they were assigned to the tier they have been assigned the longest. Five, nine and seven regions were assigned to the yellow, orange, and red tier, respectively (Table S1).

### Data Analysis

Based on the ten indicators of drinking, sub-groups of individuals characterized by common patterns of multiple alcohol use behaviors were identified using LCA. Following established recommendations (Lanza et al., 2011), a series of statistical models were estimated. Testing time (cohorts in our case) invariance in LCA is crucial to ensure the comparability of classes and draw valid conclusions about change in their prevalence over time (Hickendorff et al., 2018). For this reason, before examination of measurement invariance, we first estimated models in the 2015–2020 combined sample and then separately within each cohort. All analyses were conducted separately by gender. Because combined samples with different weighting scores were used, non-weighted data were used at this stage. For a description of the statistical (absolute and relative model fit indices) and conceptual standards used to compare the different profile solutions (see Sorgente et al. (2019). To provide support to the validity of the classes, the pseudo-class method was used to test differences in coping drinking motives and rates of experiencing alcohol-related consequences in the 2015 cohort (Clark & Muthen, 2009). Lastly, the three-step procedure was used to test the effect of lockdown tiers on class membership probabilities (Asparouhov & Muthén, 2014). Analyses were performed in MPlus 7.11 using the robust maximum likelihood estimator.

## Results

Descriptive statistics on the proportion of participants who reported each alcohol use behavior, separately by cohort, are reported in Table S2.

### Identification of Latent Classes of Alcohol Use Behaviors

We compared models with two to six latent classes. As seen in Table 2, for both women and men, each of the relative fit indices (CAIC and ssBIC) decreased with each additional solution. The other fit indices did not provide clear evidence to support either the five or six-class model, except that the six-class solution exhibited the highest value of correct model probability (cmP) and presented a number of standardized residual lower than |3|. For these reasons, the six-model solution was examined first. Inspection of item response probabilities of this model, however, revealed that latent classes were not clearly distinguished (Table S3). On the other hand, classes of the five-class model were relatively distinguishable and interpretable, and entropy was higher than the six-class model and above acceptability thresholds (> 0.70). Thus, this model was deemed the best-fitting, most interpretable and most parsimonious solution to the data.

**Table 2:** Model fit statistics by gender for Latent Class Analysis models with two to six latent classes (both cohorts)

Model	*− LL*	SCF	χ2 *LRT* (*p* value)	Stdres (%)	LMR- LRT (*p* value)	BLRT	CAIC	ssBIC	BF	cmP	SSS	Entropy
*Women*												
Two-class	− 18,486.365	1.30	15,478.245 (*p* > .001)	19.71	7284.164 (*p* < 0.001)	< 0.001	37,014.731	37,149.763	0.00	0.000	615	0.944
Three-class	− 16,114.820	1.05	4212.802 (*p* > .001)	20.19	4692.487 (*p* < 0.001)	< 0.001	32,293.640	32,499.404	0.00	0.000	498	0.986
Four-class	− 15,536.334	1.03	1947.927 (*p* > .001)	9.62	1144.629 (*p* < 0.001)	< 0.001	31,158.668	31,435.162	0.00	0.000	437	0.887
Five-class	− 15,441.485	1.07	1723.971 (*p* > .001)	7.21	187.674 (*p* < 0.001)	< 0.001	30,990.970	31,338.196	0.00	0.000	138	0.884
Six-class	− 15,358.161	1.10	1527.773 (*p* > .001)	2.88	164.869 (*p* < 0.05)	< 0.001	30,846.323	31,264.280	Na	1.000	129	0.862
*Men*												
Two-class	− 13,003.030	1.08	13,371.401 (*p* < 0.001)	24.87	7481.301 (*p* < 0.001)	< 0.001	26,048.061	26,174.903	0.00	0.000	657	0.971
Three-class	− 11,815.458	1.02	3842.066 (*p* < 0.001)	20.30	2348.590 (*p* < 0.001)	< 0.001	23,694.915	23,888.199	0.00	0.000	305	0.991
Four-class	− 11,433.833	1.03	1792.755 (*p* < 0.001)	10.15	754.716 (*p* < 0.001)	< 0.001	22,953.665	23,213.391	0.00	0.000	305	0.907
Five-class	− 11,298.985	1.04	1550.583 (*p* < 0.001)	7.61	266.680 (*p* < 0.001)	< 0.001	22,705.970	23,032.137	0.00	0.000	289	0.905
Six-class	− 11,183.159	1.04	1091.158 (*p* < 0.01)	3.05	164.869 (*p* < 0.05)	< 0.001	22,496.319	22,888.927	Na	1.000	288	0.851

*−* *LL* log likelihood, *SCF* scaling correction factor of the robust maximum likelihood estimator, *χ2 LRT* likelihood ratio chi square goodness-of-fit; Stdres = standardized residuals, *LMR-LRT* Lo–Mendell–Rubin likelihood ratio test, *BLRT* bootstrapped likelihood ratio test, *CAIC* Consistent Akaike information criterion, *ssBIC* sample-size adjusted Bayesian information criterion, *BF* Bayesian factor, *cmP* approximate correct model probability, *SSS* smaller class numerosity

As shown in Table 3, the model was fully invariant across cohorts for women and partially invariant for men (one parameter let free to vary). Table 4 presents the results of the final five-class model by gender. The item response probabilities represent the likelihood that the participants in each latent class reported exhibiting a specific alcohol use behavior. Examination of item response probabilities confirmed the profiles in large part reflected the drinking statuses found in previous studies^1^ (e.g., Aresi et al., 2020). Three classes were confirmed: *current non-drinker class* (CND), *weekend risky* (WRD) and *weekend non-risky drinkers* (WnRD). However, we found that those characterized by high probabilities of drinking on all seven days of the week were distinguished into two subgroups: *daily non-risky* (DnRD) and *daily risky drinkers* (DRD), depending on whether they were likely to report any of the two risky drinking behaviors (i.e., drunk in the past month or HED). In sum, we found one class characterized by abstention, two by moderate and two by risky drinking patterns. In the 2015 cohort, one-way analyses of variance and post-hoc analyses showed that, in both genders, DRDs reported the greatest coping drinking motives [women: *F*(4, 3108) = 126.541, *p* < 0.001; men: *F*(4, 2010) = 134.738, *p* < 0.001] and the greatest number of alcohol-related negative consequences [women: *F*(4, 3,108) = 236.944, *p* < 0.001; men: *F*(4, 2010) = 185,655, *p* < 0.001] as compared to WRDs and the other more moderate drinking patterns, thus adding to the conceptual validity of the classes (Figures S1 and S2).

**Table 3:** Chi-square difference tests based on log likelihood values

	− LL	SCF	*d*	Δ	*df*	*p-value*
*Women*						
Baseline model	− 17,607.41	1.04	109			
Full invariance	− 17,633.33	1.06	59	25.92	50	0.441
*Men*						
Baseline model	− 13,083.84	1.03	109			
Full invariance	− 13,133.47	1.06	59	49.64	50	< 0.001
Partial invariance	− 13,129.61	1.06	60	3.87	49	0.999

*LL* model log likelihood, *SCF* scaling correction factor of the robust maximum likelihood estimator; *d* = number of free parameters; Δ = difference test value; d*f* = degree of freedom of the difference test

**Table 4:** Item-response probabilities for five-class LCA model, by gender (both cohorts)

	Latent Class				
	CND	WnRD	WRD	DnRD	DRD
*Women*					
Any drink in past month	0.00	**1.00**	**1.00**	**1.00**	**1.00**
Any drink Monday	0.00	0.01	0.08	**0.99**	**0.95**
Any drink Tuesday	0.00	0.01	0.12	**0.99**	**0.98**
Any drink Wednesday	0.00	0.04	0.15	**0.99**	**0.97**
Any drink Thursday	0.00	0.03	0.22	**1.00**	**1.00**
Any drink Friday	0.00	0.35	**0.66**	**1.00**	**0.99**
Any drink Saturday	0.00	**0.92**	**0.94**	**1.00**	**0.99**
Any drink Sunday	0.00	0.49	**0.64**	**0.97**	**0.94**
Drunk in past month	0.01	0.01	**0.54**	0.12	**0.80**
Past month HED	0.02	0.01	**0.54**	0.06	**1.00**
*Men*					
Any drink in past month	0.00	**1.00**	**1.00**	**1.00**	**1.00**
Any drink Monday	0.00	0.03	0.18	**0.97**	**0.98**
Any drink Tuesday	0.00	0.04	0.09	**1.00**	**0.99**
Any drink Wednesday	0.00	0.09	0.17	**0.98**	**0.99**
Any drink Thursday	0.00	0.08	0.20	**1.00**	**0.99**
Any drink Friday	0.00	0.44	**0.62**	**1.00**	**0.99**
Any drink Saturday	0.00	**0.94**	**0.90**	**1.00**	**1.00**
Any drink Sunday	0.00	**0.54**	**0.70**	**0.98**	**0.96**
Drunk in past month	0.04	0.05	**0.77**	0.00	**0.77**
Past month HED	0.03	0.02	0.69*	0.00	**0.80**

*CND* current non-drinkers, *WnRD* weekend non-risky drinkers, *WRD* weekend risky drinkers, *DnRD* daily non-risky drinkers, *DRD* daily risky drinkers. Bold values indicate class-defining probabilities (> 0.50). *Parameter let free in invariance tests across cohorts

### Assessing Changes in Class Prevalence Across Cohorts

**Fig. 1:**
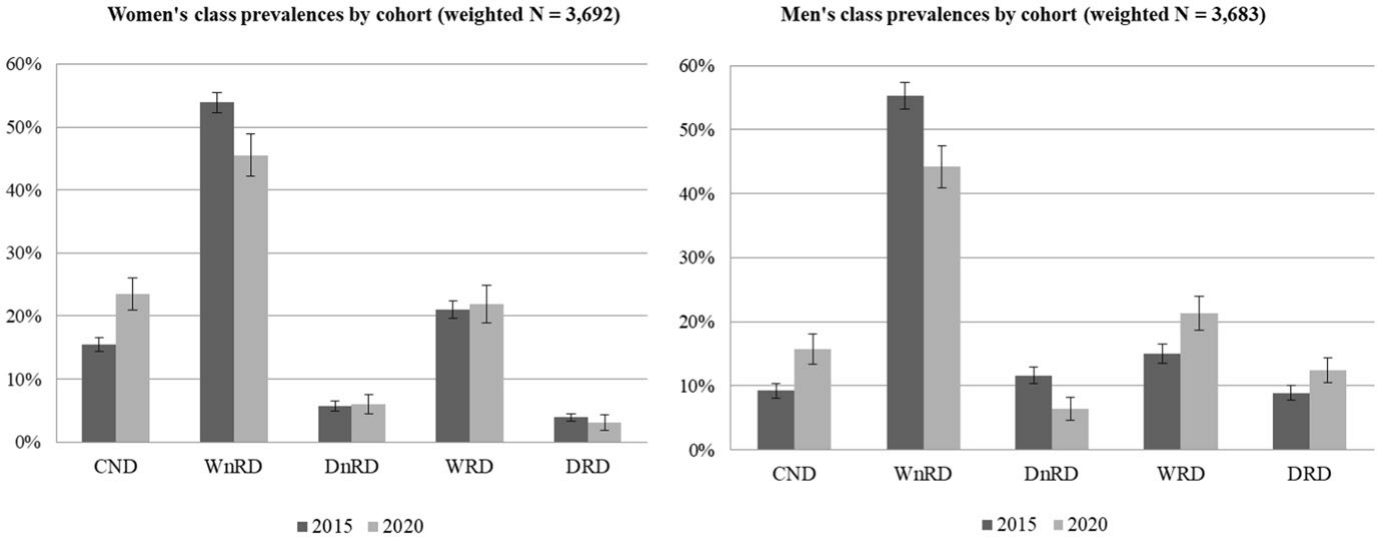
Drinker class prevalences (95% C.I.) in 2015 and 2020, by gender. *Note*: Ns represent weighted sample numerosity. 95% confidence intervals are displayed. CND = current non-drinkers; WnRD = weekend non-risky drinkers; WRD = weekend risky drinkers; DnRD = daily non-risky drinkers; DRD = daily risky drinkers

We compared class prevalence rates across 2015 and 2020 cohorts. For both female (*p* < 0.01) and male (*p* < 0.001) participants, results indicated non equivalence. We then saved participants’ most likely class membership and calculated 2015 and 2020 weighted class prevalence rates. Ninety-five per cent confidence intervals were used to determine any variation across cohort (Fig. 1). Among women, CNDs substantially increased from 15.5% in 2015 to 23.5% in 2020, whereas WnRDs decreased from 53.9% to 45.5%. However, prevalence rates of the other three classes did not vary significantly across cohorts. Similarly, the proportion of men assigned to the CND class increased from 9.2% in 2015 to 15.7% in 2020, and membership in the WnRD class decreased from 55.3% to 44.2%. There were also differences in the prevalence of the other three classes. DnRDs decreased by about half from 11.6 to 6.4%, whereas both WRDs and DRDs increased from 15.0 to 21.3% and 8.9 to 12.4%, respectively.

Lastly, lockdown tier allocation was included in the model to test its impact on 2020 class membership (Table 5). For both genders, no significant association between latent class membership and lockdown tiers was found (all ps > 0.05).

**Table 5:** Associations between latent class membership and lockdown tiers by gender

	Women				Men			
	CND OR (95% CI)∗∗	WnRD OR (95% CI)∗∗	DnRD OR (95% CI)∗∗	WRD OR (95% CI)∗∗	CND OR (95% CI)∗∗	WnRD OR (95% CI)∗∗	DnRD OR (95% CI)∗∗	WRD OR (95% CI)∗∗
Lockdown tier (orange and red)	1.52 (0.58, 4.00)	1.71 (0.68, 4.32)	1.26 (0.43, 3.74)	1.33 (0.51, 3.45)	1.20 (0.63, 2.29)	1.59 (0.90, 2.79)	1.48 (0.73, 3.01)	1.25 (0.63, 2.47)

All comparisons are with reference class DRD. *CND* current non-drinkers, *WnRD* weekend non-risky drinkers, *WRD* weekend risky drinkers, *DnRD* daily non-risky drinkers, *DRD* daily risky drinkers

**Odd ratios with 95% confidence limits that do not include 1 can be considered to reflect a significant group difference

## Discussion

This study used a person-centered approach to compare patterns of alcohol use among Italian young adults in 2015 and during the November 2020 COVID-19 lockdown. The use of LCA to identify subgroups of different drinking patterns allowed us to simultaneously test the *Availability* and *Stress and Coping hypotheses* on the effects of this pandemic on alcohol use (Rehm et al., 2020). Our findings are broadly consistent with results of other studies (Busse et al., 2021; Garnett et al., 2021; Sallie et al., 2020) and indicate there have been changes in the prevalence of drinking patterns before and during the pandemic, though these changes are different across genders.

Among both men and women, we found substantial increases in the proportion of individuals who were almost entirely abstaining from alcohol (i.e., *Current non-drinkers*) paired with decreases in the proportion of those who drink moderately over the weekend (i.e., *Weekend non-risky drinkers*) (Hp1). These results offer support to the *Availability hypothesis*: in Italy’s “wet” drinking culture (Aresi et al., 2018, 2020; Beccaria, 2010; Room, 2007), the strongest effect was that reduced opportunities to drink apparently led to decreases in alcohol use during the lockdown in the general young adult population and namely among those who drank moderately (Rehm et al., 2020).

Among men only, however, our results suggest a polarization effect whereby the proportion of moderate drinkers (i.e., *Weekend and Daily non-risky drinkers*) decreased, whereas the proportion of risky drinkers (i.e., *Weekend and Daily risky drinkers*) increased from 2015 to during the pandemic in 2020 (Hp2). Given these two latter groups were characterized by the greatest coping drinking motives, this result provides support to a self-medication use of alcohol under the *Stress and Coping hypothesis* among heavy drinking men. This result is novel and casts light on the gendered stress—alcohol misuse relation during the pandemic (Rodriguez et al., 2020; Schmits & Glowacz, 2021). Importantly, the 40% increase (from almost 9 to 12.5 per cent) in young men drinking on all seven days of the week and engaging in risky drinking (DRD) is particularly alarming. This group was more likely to use alcohol as a coping mechanism and displayed a disproportionate number of alcohol-related negative consequences as compared to any other group. Previous studies have demonstrated that, among young Italians, extending consumption beyond the typical Thursday—Sunday pattern does not denote problematic alcohol use per se, as it is in other countries, nor bears negative implications for wellbeing (Aresi et al., 2020; Cleveland et al., 2012; Piumatti et al., 2019). However, as in the case of DRDs, this pattern becomes concerning and is socially censored when paired with episodes of drunkenness and heavy drinking sessions (Aresi & Pedersen, 2016; Hoeppner et al., 2012). Therefore, these individuals may not only suffer from the health consequences of heavy alcohol use, but also incur social rejection, which may further push them toward solitary drinking and exacerbate their psychological distress even after the lockdown is lifted. Consistently, previous findings from past epidemics (i.e., SARS) suggest increases in substance use to cope may persist beyond the pandemic (Wu et al., 2009). For all these reasons, this group should be the target of selective prevention efforts and treatment that provides psychological support that extends beyond substance use. Traditional in-person interventions may become difficult to implement in times of quarantine; thus, health interventions that can be delivered at a distance may be pursued instead. In this regard, psychological interventions delivered via computers or mobile applications have been recognized as efficient and effective strategies of addiction treatment (Giroux et al., 2017).

From a prevention standpoint, lockdowns may represent unique opportunities to re-evaluate one’s relationship with alcohol (Nicholls & Conroy, 2021). This applies not only to those who drink heavily but to all drinkers who may make changes to their drinking practices towards healthier behaviours. To this end, interventions that encourage moderate alcohol use and that target the general population may be beneficial. Such efforts may be especially effective for both women and men who engage in moderate drinking behaviours. There is growing evidence that online and mobile interventions, such as computerised serious educational games (Rodriguez et al., 2014) and personalized feedback interventions (Kohl et al., 2013), can have positive effects on alcohol use and other health-related behaviours. Given the distinct patterns of alcohol use among the identified classes, our findings support the value of such personalised interventions and point to the need for future research that examines their effectiveness across the latent classes.

Lastly, contrary to our expectations (Hp3), increasingly restrictive lockdown tiers were not associated with increased likelihood to belong to lighter drinking patterns. We speculate this is because on one side alcohol availability was greatly limited in all tiers (i.e., the least restrictive tier meant drinking outlets would close at 6 p.m.), and on the other because other sources of alcoholic beverages (e.g., off-premise and delivery) remained easily accessible throughout the lockdown. These countervailing effects may explain why our results are not consistent with other that introducing policy to reduce alcohol availability (e.g., excluding alcoholic beverages from the list of essential items sold in supermarkets) or restrictive marketing practices bear public health benefits by reducing alcohol use in the population and alleviating pressure on the healthcare system (Reuter et al., 2020; Stockwell et al., 2021).

Limitations of the current study suggest avenues for future research. First, the repeated cross-sectional design constrains the interpretation of causal effects (e.g., lockdown measures determined change in alcohol use patterns) nor allows determining the direction of the transitions from and to drinking patterns. In addition, because the two cohorts were five years apart, secular trends of reduced alcohol intake among young people may at least in part explain the differences we found. We also note there were inconsistencies in how data were collected in the two surveys, the main being the time frame (past three months Vs. one month) of a typical week for responding to some alcohol use indicators. The degree of bias introduced by such inconsistency is likely to be limited given the three-month time frame does not include periods that are known for seasonal fluctuations in alcohol use (namely summer and winter breaks). Further longitudinal and mixed-method research is needed to better examine causal relationships, assess changes in further waves and restrictions, and get a deeper understanding of these issues. An additional limitation is that the present study employed only self-report measures, which might be susceptible to response bias.

### Conclusions and Implications for Research and Practice

This study contributes to the literature on alcohol use during the COVID-19 health emergency. Results indicate gender-specific changes in the prevalence of the five drinker profiles. In support of the *Availability hypothesis*, increases in abstaining and moderate drinker classes were observed among both young women and young men. From a public health perspective, however, declines in alcohol consumption exhibited by most youth should not obscure the finding that, among men, there were also increases in the prevalence of patterns characterized by heavy risky drinking to cope with stress (*Stress and Coping hypothesis*). Accordingly, it is important for policy makers, prevention scientists and practitioners, and outreach health services to focus on those who drink daily and heavily. Future research should investigate whether the prevalence (> 10%) of this group of young men stabilized or have returned to usual levels as lockdown restrictions were lifted and people resumed their normal lives.

An unexpected result was that drinking classes were unaffected by differences in the limitation to the operation of drinking outlets across Italian regions. This is likely because, even under the stricter rules, alcoholic beverages were easily accessible across a variety of sites and through delivery as it is in usual times. Finally, we suggest that our study makes an important methodological contribution to the field. Our use of LCA allowed developing a multifaceted and thorough portrait of alcohol use patterns during a public health emergency. We recommend LCA as an important tool for future studies in this field.

## Supplementary Information

Below is the link to the electronic supplementary material.

Supplementary file1 (DOCX 144 kb)

## Data Availability

The data underlying this article were provided by the ©Osservatorio Giovani of the Istituto Toniolo di Studi Superiori and will be shared with permission of the Osservatorio. Requests to access the datasets should be directed to the corresponding author.

## Footnotes

1 The four drinking classes were: those who have not been drinking recently (Current Non-Drinkers—CND), two classes of weekend drinkers distinguished by the probability of engaging in risky drinking (Weekend Non-Risky Drinkers – WNRD, and Weekend Risky Drinkers—WRD), and one class of Daily Drinkers (DD).
