# Supplementary material for: Patterns of Alcohol Use Among Italian Young Adults Before and During a COVID-19 Lockdown: A Latent Class Analysis Study
Source: J Prev (2022). 2022 Mar 19;43(2):191–208. doi: 10.1007/s10935-022-00675-2 (PMC8934024; doi:10.1007/s10935-022-00675-2)
Supplement: Supplementary file 1 — Supplementary file1 (DOCX 144 kb) [file 10935_2022_675_MOESM1_ESM.docx]

**Table S1**. Tier assignment of the 19 Italian regions and two autonomous provinces.

|  | Week | | |  |
| --- | --- | --- | --- | --- |
| Region/autonomous province | 06-13/11/2020 | 14-20/11/2020 | 21-27/11/2020 | Assigned tier |
| Lombardy | Red | Red | Red | Red |
| Piedmont | Red | Red | Red | Red |
| Calabria | Red | Red | Red | Red |
| Valle d’Aosta | Red | Red | Red | Red |
| Puglia | Orange | Orange | Orange | Orange |
| Sicily | Orange | Orange | Orange | Orange |
| Abruzzo | Yellow | Orange | Red | Orange |
| Basilicata | Yellow | Orange | Orange | Orange |
| Campania | Yellow | Red | Red | Red |
| Emilia-Romagna | Yellow | Orange | Orange | Orange |
| Friuli-Venezia Giulia | Yellow | Orange | Orange | Orange |
| Lazio | Yellow | Yellow | Yellow | Yellow |
| Liguria | Yellow | Orange | Orange | Orange |
| Marche | Yellow | Orange | Orange | Orange |
| Molise | Yellow | Yellow | Yellow | Yellow |
| Trento a.p. | Yellow | Yellow | Yellow | Yellow |
| Bolzano a.p. | Yellow | Red | Red | Red |
| Sardinia | Yellow | Yellow | Yellow | Yellow |
| Tuscany | Yellow | Red | Red | Red |
| Umbria | Yellow | Orange | Orange | Orange |
| Veneto | Yellow | Yellow | Yellow | Yellow |

Note. a.p. = autonomous province.

Table S2. *Proportion of respondents reporting drinking behaviors, by cohort and gender*.

|  | | | |
| --- | --- | --- | --- |
| Alcohol behavior | 2015  (*N* = 5,950) | 2020  (*N* = 1,736) | *χ2* |
| Drink in last month | 87.9 | 83.4 | 23.965*** |
| Typically drink on Monday | 15.9 | 17.8 | 3.423 |
| Typically drink on Tuesday | 16.6 | 17.4 | 0.602 |
| Typically drink on Wednesday | 19.4 | 20.7 | 1.531 |
| Typically drink on Thursday | 20.2 | 20.6 | 0.099 |
| Typically drink on Friday | 46.7 | 48.4 | 1.663 |
| Typically drink on Saturday | 82.3 | 78.0 | 16.833*** |
| Typically drink on Sunday | 54.2 | 51.2 | 4.859* |
| Been drunk in last month | 19.6 | 19.4 | 0.034 |
| HED in last two weeks | 17.4 | 23.6 | 32.680*** |
| Note: Values indicate % reporting the behavior, *N* = sample size; HED, heavy episodic drinking; BAC, blood alcohol content. **p* < 0.05, ***p*< 0.01, ****p*< 0.001. | | | |

**Table S3**. *Item-response probabilities and class prevalence rates for six-class LCA model, by gender (both cohorts).*

|  | **Latent Class** | | | | | |
| --- | --- | --- | --- | --- | --- | --- |
|  | Women | | | | | |
|  | 1 | 2 | 3 | 4 | 5 | 6 |
| Any drink in past month | 1.00 | 1.00 | 1.00 | 0.00 | 1.00 | 1.00 |
| Any drink Monday | 0.96 | 1.00 | 0.16 | 0.00 | 0.02 | 0.01 |
| Any drink Tuesday | 0.98 | 1.00 | 0.26 | 0.00 | 0.00 | 0.01 |
| Any drink Wednesday | 0.97 | 1.00 | 0.30 | 0.00 | 0.02 | 0.04 |
| Any drink Thursday | 1.00 | 1.00 | 0.41 | 0.00 | 0.04 | 0.02 |
| Any drink Friday | 0.99 | 1.00 | 0.79 | 0.00 | 0.51 | 0.35 |
| Any drink Saturday | 0.99 | 1.00 | 0.92 | 0.00 | 0.95 | 0.92 |
| Any drink Sunday | 0.93 | 0.98 | 0.66 | 0.00 | 0.61 | 0.49 |
| Drunk in past month | 0.95 | 0.11 | 0.43 | 0.01 | 0.63 | 0.00 |
| Past month HED | 1.00 | 0.14 | 0.42 | 0.02 | 0.64 | 0.00 |
| **Estimated Prevalence** | 2.8% | 6.4% | 12.4% | 15.3% | 12.6% | 50.5% |
|  |  | | | | | |
|  | Men | | | | | |
| Any drink in past month | 1.00 | 1.00 | 1.00 | 1.00 | 1.00 | 0.00 |
| Any drink Monday | 0.14 | 0.98 | 0.01 | 0.99 | 0.19 | 0.00 |
| Any drink Tuesday | 0.17 | 0.99 | 0.00 | 1.00 | 0.07 | 0.00 |
| Any drink Wednesday | 0.26 | 0.99 | 0.04 | 0.99 | 0.16 | 0.00 |
| Any drink Thursday | 0.34 | 0.99 | 0.00 | 1.00 | 0.17 | 0.00 |
| Any drink Friday | 0.75 | 1.00 | 0.36 | 1.00 | 0.55 | 0.00 |
| Any drink Saturday | 0.94 | 1.00 | 0.94 | 1.00 | 0.88 | 0.00 |
| Any drink Sunday | 0.67 | 0.95 | 0.51 | 0.99 | 0.71 | 0.00 |
| Drunk in past month | 0.21 | 0.85 | 0.07 | 0.04 | 0.85 | 0.04 |
| Past month HED | 0.06 | 0.84 | 0.04 | 0.07 | 1.00 | 0.03 |
| **Estimated Prevalence** | 12.4% | 9.3% | 48.4% | 10.0% | 9.9% | 9.8% |

**Figure S1**. *Mean scores of coping drinking motives by gender and drinker class.*


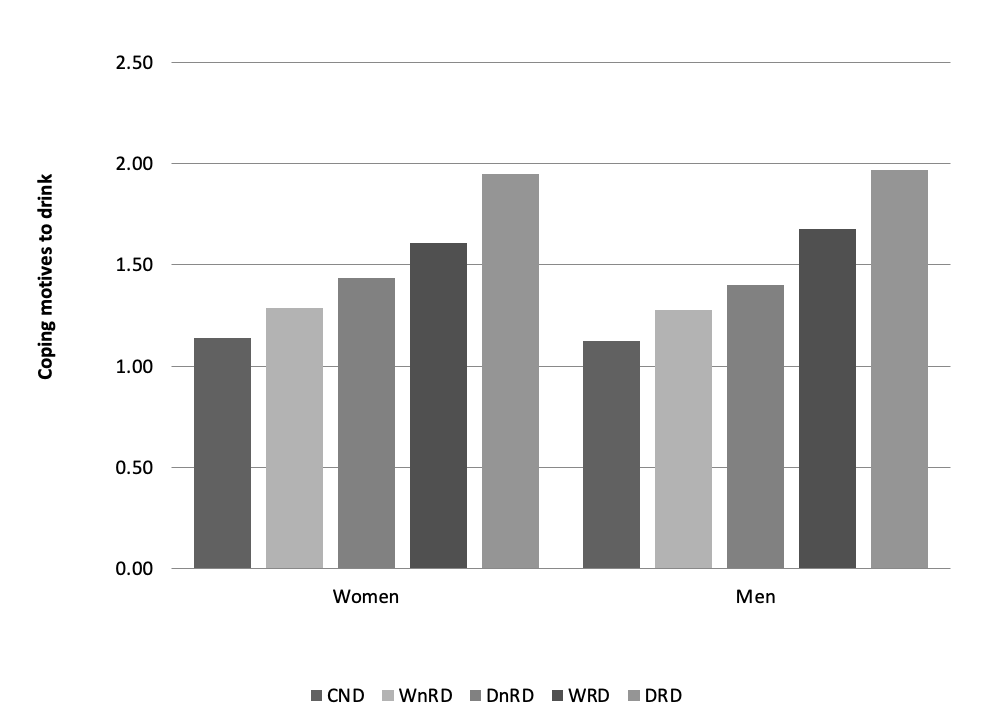


***

***

***

***

***

***

***

Note: Results of pairwise post-hoc analyses. **p* < 0.05, ***p*< 0.01, ****p*< 0.001.

Note. CND = current non-drinkers; WnRD = weekend non-risky drinkers; WRD = weekend risky drinkers; DnRD = daily non-risky drinkers; DRD = daily risky drinkers. Results of pairwise post-hoc analyses. **p* < 0.05, ***p*< 0.01, ****p*< 0.001.

**Figure S2**. *Mean number of reported alcohol-related consequences by gender and drinker class.*


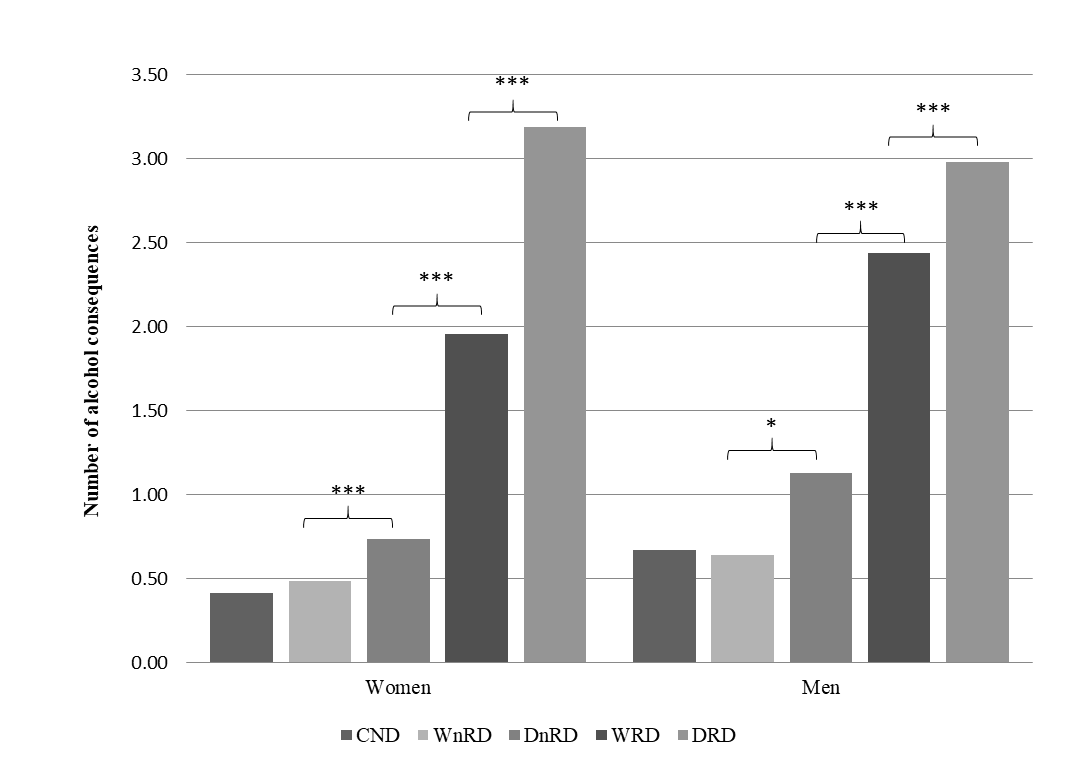


Note. CND = current non-drinkers; WnRD = weekend non-risky drinkers; WRD = weekend risky drinkers; DnRD = daily non-risky drinkers; DRD = daily risky drinkers. Results of pairwise post-hoc analyses. **p* < 0.05, ***p*< 0.01, ****p*< 0.001.
